# Bmi1 inhibitor PTC-209 promotes Chemically-induced Direct Cardiac Reprogramming of cardiac fibroblasts into cardiomyocytes

**DOI:** 10.1038/s41598-020-63992-8

**Published:** 2020-04-28

**Authors:** Gianluca Testa, Michele Russo, Giorgia Di Benedetto, Matteo Barbato, Silvia Parisi, Flora Pirozzi, Carlo Gabriele Tocchetti, Pasquale Abete, Domenico Bonaduce, Tommaso Russo, Fabiana Passaro

**Affiliations:** 1 Department of Medicine and Health Sciences “Vincenzo Tiberio”, University of Molise, Via Francesco de Sanctis 1, 86100 Campobasso, Italy; 2 Department of Translational Medical Sciences, University of Naples “Federico II”, Via Sergio Pansini 5, 80131 Napoli, Italy; 3 Department of Molecular Medicine and Medical Biotechnology, University of Naples “Federico II”, Via Sergio Pansini 5, 80131 Napoli, Italy

**Keywords:** Regenerative medicine, Reprogramming, Transdifferentiation

## Abstract

The development of therapeutic approaches based on direct cardiac reprogramming of fibroblasts into induced-cardiomyocytes (iCM) has emerged as an attractive strategy to repair the injured myocardium. The identification of the mechanisms driving lineage conversion represents a crucial step toward the development of new and more efficient regenerative strategies. To this aim, here we show that pre-treatment with the Bmi1 inhibitor PTC-209 is sufficient to increase the efficiency of Chemical-induced Direct Cardiac Reprogramming both in mouse embryonic fibroblasts and adult cardiac fibroblasts. PTC-209 induces an overall increase of spontaneously beating iCM at end-stage of reprogramming, expressing high levels of late cardiac markers Troponin T and myosin muscle light chain-2v. The inhibition of Bmi1 expression occurring upon PTC-209 pre-treatment was maintained throughout the reprogramming protocol, contributing to a significant gene expression de-regulation. RNA profiling revealed that, upon Bmi1 inhibition a significant down-regulation of genes associated with immune and inflammatory signalling pathways occurred, with repression of different genes involved in interleukin, cytokine and chemokine pathways. Accordingly, we observed the down-regulation of both JAK/STAT3 and MAPK/ERK1-2 pathway activation, highlighting the crucial role of these pathways as a barrier for cardiac reprogramming. These findings have significant implications for the development of new cardiac regenerative therapies.

## Introduction

The adult mammalian heart is unable to fully restore cardiac function after injury, due to the lack of endogenous repair mechanisms^1,2^. Thus, the development of alternative approaches to regenerate and repair the injured myocardium is considered a top priority in treating heart failure^3,4^.

Direct cardiac reprogramming (DCR) of fibroblasts into induced CMs (iCMs) has emerged as an attractive strategy. Since the first attempt based on retroviral delivery of the pivotal cardiac transcription factors (TFs) Gata4, Mef2c, and Tbx5 (G-M-T)^5^, alternative sets of reprogramming factors based on different TFs combinations^6,7^ or microRNAs^8,9^, alone or with small chemical compounds capable to inhibit specific signalling pathways or enzymes involved in epigenetic modifications, have been reported^4,10^.

Recent work^11^ demonstrated that a solo chemical compound cocktail was able to functionally replace ectopic expression of TFs. The cocktail, comprising six molecules including CHIR99021 (C- a GSK3 inhibitor), RepSox (R- a TGFβR1 inhibitor), Forskolin (F- which sustains cAMP synthesis), Valproic Acid (VPA - a HDAC inhibitor), Parnate (P- an inhibitor of lysine-specific demethylase 1) and TTNPB (T- a highly selective retinoic acid analogue) and thus named CRFVPT, could induce beating clusters of cardiac cells from mouse fibroblasts *in vitro*^11^ and *in vivo*^12^, although with low efficacy. A similar Chemical-induced Direct Cardiac Reprogramming (CiDCR) has also been reported in human fibroblasts with a combination of nine compounds, in part overlapping the cocktail used to reprogram mouse fibroblasts^13^.

Although the precise mechanisms underlying CRFVPT activity remain unclear, it can be argued that the two epigenetic modulators Parnate and VPA can help to break through the epigenetic obstacles existing in different cell types, while the two “mesenchymal to epithelial transition” modulators CHIR99021 and RepSox suppress the phenotype of the starting cell. Finally, Forskolin and TTNPB somehow induce the characteristics of the designated cells.

The increase of the efficiency of the reprogramming progress due to the interventions on the epigenetic profile has prompted new strategies to improve the whole process.

Recently, the investigation of epigenetic dynamics accompanying DCR by TFs revealed that H3K27me3 and H3K4me3 undergo an early redistribution at cardiac loci and late alterations at fibroblast loci^14^, with the subsequent activation of the cardiac program and suppression of the fibroblast phenotype. Similar changes are needed for the induction of cardiac gene expression during the microRNA-induced reprogramming process^15^. Moreover, the timing of histone methyltransferase inhibition is critical for its effects on reprogramming. In fact, it has been reported that late inhibition of the methyltransferase G9a, responsible for the H3K9me1/2 methylation, increases reprogramming efficiency of fibroblasts^16^ while pre-treatment with a G9a inhibitor reduced reprogramming efficiency^17^, demonstrating that specific compound administration is effective only at definite timeframes to increase reprogramming efficiency^16^. Nevertheless, how the handling of universal epigenetic regulators affects the core gene regulatory network in a specific cell type is still largely unknown. A deeper comprehension of these interactions will allow the identification of appropriate regulators involved in lineage reprogramming.

The identification and modulation of target molecules involved in lineage conversion represents a major challenge^5–7^. To address this question, Zhou *et al*. performed a screening for epigenetic regulators with a significant role in iCM generation^18^ and found that reprogramming efficiency of G-M-T was significantly enhanced by the knockdown of the essential component of the polycomb repressive complex 1 (PRC1), Bmi1. The silencing of Bmi1 by shRNAs de-repressed the activity of Gata4 during the reprogramming process replacing the need of exogenous Gata4 during the process^18^. Interestingly, the positive effect provoked by Bmi1 knockdown was confirmed as early as three days after viral transduction and was effective only when the vector was administered early in the iCM reprogramming process^18^.

Since the use of integrative viruses, frequently adopted in DCR approaches, is related to elevated risks of oncogenesis and genomic disruption, the adoption of a strategy based on the activity of small molecules to induce trans-differentiation via non-genetic strategies provides substantial foundation for pharmacological interventions to be translated into the clinic^10^.

On this basis, our work aimed at increasing the efficacy of CiDCR by modulating Bmi1 expression with a known inhibitor, PTC-209^19^. We found that 24 hours pre-treatment with PTC-209 is sufficient to increase the efficiency of CiDCR both in mouse embryonic fibroblasts (MEFs) and adult (5 weeks old) cardiac fibroblasts (CFs). Bmi1 downregulation induced by pre-treatment with 1 μM PTC-209 significantly enhances iCMs generation compared to untreated cells, providing evidences that not only the targeting molecules, but also the timing of inhibition is crucial for its effect on CiDCR efficacy. The expression profile of CFs pre-treated with PTC-209 revealed the down-regulation of chemokines, interleukins and cytokines associated with immune and inflammatory signalling pathways. Accordingly, signalling converging on JAK/STAT3 and MAPK/ERK1-2 activation resulted in compromised reprogramming efficiency. These results could pave the way to the identification of new targeting molecules and novel strategies to booster the current efficiency of CiDCR toward iCM formation.

## Results

### Pre-treatment with PTC-209 for 24 h enhances CiDCR efficiency of MEFs and adult CFs

Downregulation of the polycomb ring finger oncogene Bmi1 has been identified as a booster in cardiac reprogramming^18^.

To explore whether CiDCR efficacy could be enhanced by inhibition of Bmi1 expression, we used PTC-209 compound, a recent and well characterized chemical inhibitor of Bmi1, which acts post-transcriptionally by down-regulating Bmi1 protein^19^.

To define the critical window for Bmi1 inhibition, we started by testing whether the addition of PTC-209 to the CRFVPT cocktail could increase the reprogramming efficacy of MEFs. To this aim, we carried out the reprogramming protocol as previously described^11^, starting from MEFs isolated from 13.5 dpc embryos of the C57BL strain. Cells were plated on Matrigel pre-coated plates one day before the addition of Cardiac Reprogramming Medium (CRM), containing the small molecule cocktails, plus PTC-209 at different concentrations. At day 16, the medium was changed into Cardiomyocyte Maintaining Medium (CMM) for at least 10 days more. Finally, the reprogramming efficiency of iCM was determined at day 24 by flow cytometry analysis, measuring the percentage of muscle α- myosin heavy chain (α -MHC) positive cells (α-MHC^+^).

We found that concurrent administration of PTC-209 and CRFVPT cocktail caused a dramatic concentration-dependent decrease in the percentage of α-MHC^+^ cells upon MEFs reprogramming, with respect to DMSO-treated cells (data not shown).

**Figure 1:**
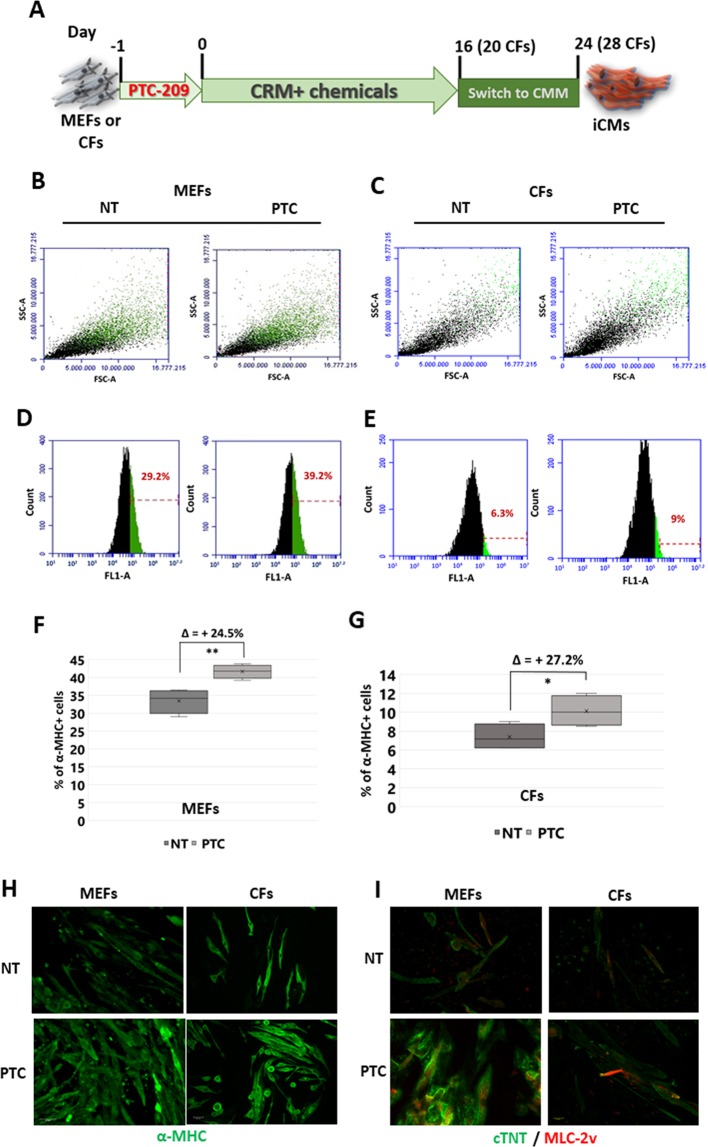
(**A**) Schematic representation of direct cardiac reprogramming protocol. MEFs or CFs were plated on Matrigel pre-coated plates in fibroblast growth medium containing 1 μM PTC-209 (PTC) or DMSO (NT) one day before the addition of Cardiac Reprogramming Medium (CRM) containing the small molecule cocktails. At day 16 for MEFs or 20 for CFs, the medium was changed into Cardiomyocyte Maintaining Medium (CMM) for at list 10 days more. Samples were analysed at final stage of reprogramming (day 24 or 28, for MEFs and CFs respectively). (**B,C**) Representative 2D scatter plots showing end-stage reprogrammed cell population upon 24 h PTC-209 pre-treatment (PTC) or DMSO (NT). Green dots represent α-MHC + cells. Typical results obtained starting from MEFs are in (**B**); those starting from CFs are in (**C**). (**D,E**) Representative plots showing fluorescent cells vs total cell counts, with the percentage of α-MHC + cells highlighted in green, at final stage of reprogramming. The increasing in α-MHC + cells upon PTC-209 pre-treatment is shown in (**D**) for MEFs and in (**E**) for CFs. (**F,G**) Box plots showing the percentage of α-MHC + cells, normalized to cell count, at final stage of reprogramming from MEFs (**F**) or CFs (**G**). For each data set: n = 4; Δ = mean of % of α-MHC + cell increase upon 24 h PTC-209 pre-treatment. *p < 0.05, **p < 0.01. (**H**) Representative immunostaining of cardiac marker α-MHC (green). (**I**) Representative immunostaining of cardiac markers MLC-2v (red) and cTNT (green). Images are related to different areas of beating clusters of iCMs at end-stage of reprogramming from MEFs and from CFs. Scale bars: 40 μM.

This prompted us to consider that, being chromatin remodelling a prerequisite to cell fate conversion, the inhibition of Bmi1 expression could be more effective if carried out before the administration of the reprogramming cocktail. On this assumption, we pre-treated MEFs for 24 h with PTC-209, and then cells were treated according to the protocol as depicted in Fig. 1A. Among different doses of PTC-209 tested, we found that pre-treatment with 1 μM PTC-209 reliably enhanced the percentage of α-MHC^+^ cells at final stage of reprogramming (Fig. 1B,D,F).

Considering that CFs are the major *in vivo* target for iCM reprogramming, we next determined the effect of PTC-209 pre-treatment on the conversion of adult (5 weeks) CFs to iCMs. Flow cytometry results indicated that the overall conversion efficiency induced by CiDCR was lower than that observed in MEFs. Nevertheless, 24 h pre-treatment with 1 μM PTC-209 was able to increase (up to 27%) the efficiency of reprogramming also of CFs (Fig. 1C,E,G).

Immunostaining revealed that iCMs derived from both MEFs or CFs were not only positive for α-MHC (Fig. 1H), but also exhibited high expression of late cardiac markers troponin-T (cTNT) and myosin light chain-2v (MLC-2v), with a clear cross-striated pattern (Fig. 1I).

Quantitative RT-PCR confirmed the expression of cardiac-specific markers, such as cTNT, Gata4, Hcn4, Myh-7b, Mef2c, Mlc-2v, Nkx2.5, Ryr2, Tbx5 and SercA4 (Supplementary Fig. S1-A and S1-B). Moreover, an increase in the number of beating clusters could be observed in pre-treated cells with respect to the NT counterpart (Supplementary Fig. S1-C). In line with these data, the percentage of α-actinin positive cells with assembled sarcomeres also increased upon PTC-209 treatment (Supplementary Fig. S1-D,E). Representative videos showing beatings areas, as well as immunostaining of isolated MEFs and CFs for fibroblast and endothelial markers are showed in Supplementary Data (Movies S1 and S2 and Supplementary Fig. S2).

These data demonstrate that 24 h pharmacological inhibition of Bmi1 is sufficient to significantly increase the efficiency of CiDCR of both MEFs and CFs, thus confirming that Bmi1 may act as an early barrier to DCR. Nevertheless, quantification of absolute number of cardiac-marker-positive iCMs at the end stage of reprogramming revealed that CiDCR efficiency varied depending on cell type assayed, suggesting intrinsic variability that should be considered to further improve the CRFVPT cocktail.

### Effects of PTC-209 pre-treatment on Bmi1 expression last throughout the reprogramming

Considering that 24 h pre-treatment with PTC-209 was sufficient to enhance the efficiency of CiDCR, we investigated the persistence of PTC-209 effects beyond the time of compound administration, throughout the whole reprogramming protocol.

**Figure 2:**
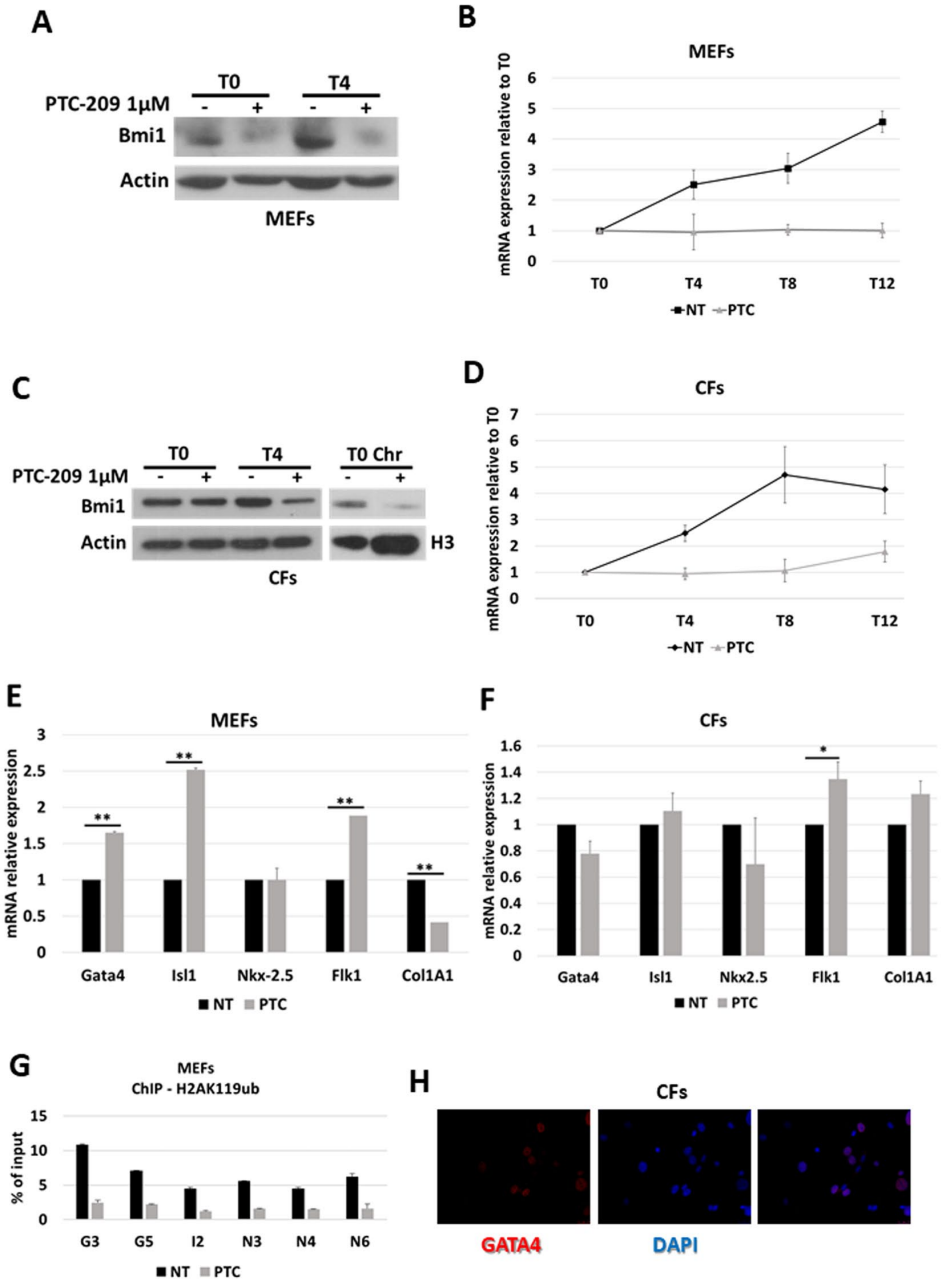
(**A,C**) Bmi1 expression profile by Western blot upon CiDCR of MEFs (**A**) or CFs (**C**) pre-treated for 24 h with 1 μM PTC-209 (PTC) or DMSO (NT), at indicated days. betaActin was used as the loading control. Panel **C** also shows Bmi1 protein levels in the chromatin fraction (Chr) of CFs at T0, upon PTC-209 pre-treatment. Histone H3 was used as loading control. White spaces between blots indicate that they were grouped from different gels or fields. (**  B,D**) Bmi1 expression profile by quantitative RT PCR on MEFs (**B**) or CFs (**D**) undergoing CiDCR with or without 24 h PTC-209 pre-treatment. For each data set, averaged numbers from biological triplicates were used for statistics. Error bars indicate mean ± SEM. (**E,F**) Expression profile of Bmi1 target genes and cardiac marker genes by quantitative RT PCR on MEFs (**E**) or CFs (**F**) upon 24 h 1 μM PTC-209 pre-treatment. For each data set, averaged numbers from biological triplicates were used for statistics. Error bars indicate mean ± SEM. *p < 0.05, **p < 0.01. (**G**) ChIP-qPCR for H2AK119ub on MEFs at Gata4 (G3 and G5), Isl1 (I2) and Nkx2-5 (N3, N4 and N6) cardiac loci. Averaged numbers from technical duplicates were used for statistics. (**H**) Representative immunostaining of Gata4 on CFs. Scale bars: 40 μM.

To this aim, we analysed the expression profile of Bmi1 in pre-treated MEFs undergoing CiDCR, in comparison to untreated cells. As expected, 24 h PTC-209 treatment induced Bmi1 down-regulation at protein levels (Fig. 2A, T0). Interestingly, this effect persisted after PTC-209 removal, coinciding with first days of CRFVPT administration (Fig. 2A, T4).

The analysis of Bmi1 expression in CFs upon PTC-209 pre-treatment showed similar results (Fig. 2C), although the downregulation of Bmi1 at T0 was not always evident in total extract. Nevertheless, immunoblot assay on fractionated cell extracts demonstrated that, upon PTC-209 pre-treatment, the amount of Bmi1 present in the fraction of proteins bound to chromatin drastically decreased (Fig. 2C).

Quantitative RT PCR analysis demonstrated that, indeed, PTC-209 pre-treatment has clear effects on Bmi1 expression throughout CiDCR of both MEFs (Fig. 2B) and CFs (Fig. 2D), counteracting the spontaneous up-regulation of Bmi1 mRNA observed in DMSO-treated cells. Of note, the increasing in Bmi1 expression upon CiDCR might indicate a possible role of PRC1 in governing molecular events in later phases of CiDCR.

These results demonstrated that the inhibition of Bmi1 expression occurring within 24 h pre-treatment with PTC-209 in both MEFs and CFs perdures after compound withdrawal. This probably causes an epigenetic perturbation, whose effects contribute to improve cellular response to CRFVPT cocktail.

Based on the data from Zhou and colleagues^18^, we wondered whether a 24 h inhibition of Bmi1 expression could be sufficient to modulate chromatin status and expression of a set of critical cardiogenic factors, including Gata4, Isl1 and Nkx2.5 or other cardiogenic markers.

As shown in Fig. 2E, the exposure of MEFs to PTC-209 induces the up-regulation of Bmi1 target genes Gata4 and Isl1, together with the increase of Flk1 expression, which is marker of cardiovascular precursor cells. Nkx2.5 expression, on the contrary, didn’t show significant modifications in agreement with previous results^18^. The increase of cardiac markers was accompanied by a concomitant down-regulation of the fibroblast marker gene Collagen 1A1 (Col1A1). ChIP qPCR of monoubiquitylation of histone H2A at lysine 119 (H2AK119ub) on cardiogenic loci demonstrated that, upon PTC-209 pre-treatment, the repressive histone mark was significantly reduced (Fig. 2G), in line with previous reports^18^. These data confirmed that in MEFs Bmi1 acts as a repressor of cardiogenic loci and its inhibition is responsible for their transcriptional activation. However, when we analysed the expression profile of cardiac markers in pre-treated CFs, we couldn’t detect the same stimulation of gene expression (Fig. 2F), as no Gata4 nor Isl1 upregulation was achieved by PTC-209 treatment. It is also important to note that Gata4 seems to be already expressed, even though at low levels, in CFs (Fig. 2H), indicating that the epigenetic status of this and probably other loci might be profoundly different between the two cell types analysed.

This prompted us to investigate the mechanisms underlying the pro-reprogramming activity of Bmi1 inhibition in CFs by addressing the PTC-209-dependent transcriptional effects.

### PTC-209 pre-treatment modulates signalling pathways governing cell fate conversion

Modifications of signalling pathways and environmental cues are expected to improve the efficiency of DCR and cardiac cell maturation. Indeed, numerous reports demonstrated the improvement of reprogramming by GMT via addition of small molecule that influence different pathways^10^.

Being Bmi1 an epigenetic modulator, its inhibition, albeit transitory, could trigger a significant perturbation of chromatin status, thus making the cell prone to trans-differentiation.

To identify the downstream targets of PTC-209 in CFs, we performed RNA-sequencing analyses of cells untreated (DMSO) or treated with 1 μM PTC-209 for 24 h and selected the most significant differentially expressed genes (DEGs).

**Figure 3:**
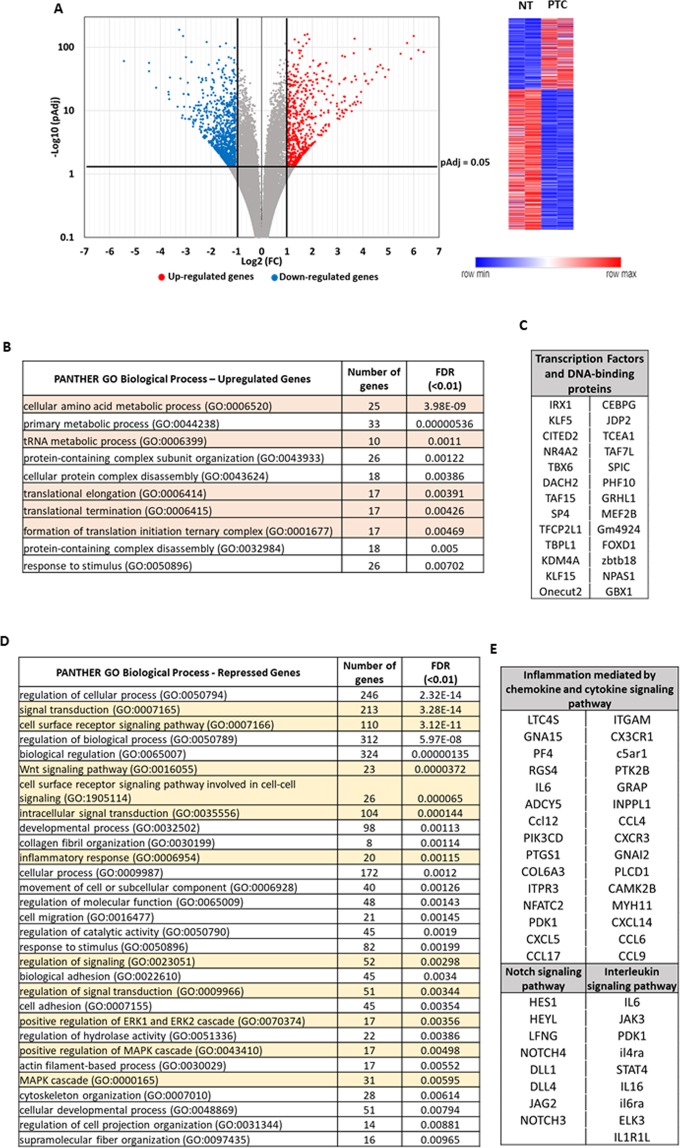
DEGs identified by RNA-seq assay in CFs pre-treated with 1 μM PTC-209 (PTC) vs DMSO (NT) cells. (**A**) Volcano plot and relative heatmap. The negative log of pAdj (base 10) is plotted on the Y-axis, and the log of the FC (base 2) is plotted on the X-axis. Red plots represent significant (pAdj < 0.05) and remarkable (FC > 2) up-regulated genes, while blue plots represent significant (pAdj > 0.05) and remarkable (FC < 2) downregulated genes. (**B**) Functional enrichment analysis of de-repressed genes for significantly over-represented Biological Process (FDR < 0.01). Highlighted in orange are the GO terms related to protein biosynthesis. (**C**) Significantly de-repressed Transcription Factors and DNA-binding proteins. (**D**) Functional enrichment analysis of repressed genes for significantly over-represented Biological Process (FDR < 0.01). Highlighted in yellow are the GO terms related to signal transduction. (**E**) Repressed genes belonging to Notch, Interleukin and inflammatory pathway, according to KEGG analysis. DEG, differentially expressed gene; FC, fold change.

Volcano plot in Fig. 3A shows the results of DEseq 2 bioinformatics analysis, which revealed 1,499 genes (569 up-regulated and 930 down-regulated, with pAdj ≤ 0.05) that were differentially expressed with a Fold Change (FC) ≥ 2 upon PTC-209 treatment. Table 1 reports the top 50 genes in both categories (see Supplementary Table S1 for complete gene list).

**Table 1:** Top 50 DEGs (pAdj < 0.05).

GeneSymbol	log2	GeneSymbol	log2
Gm19410	6.39	GJB3	-5.44
EXTL1	6.19	PPL	-4.44
CTH	6.00	GJB5	-4.44
TENM4	5.90	GJB4	-4.17
Car6	5.74	Ms4a6b	-3.68
CYB5R2	5.49	KIF26B	-3.67
COX6A2	5.01	aldh3a1	-3.65
TNFRSF19	4.87	TNS4	-3.62
SH2D6	4.78	C1QA	-3.32
adm2	4.69	LRRN4	-3.26
SOAT2	4.59	CDC42EP2	-3.21
RAB39B	4.54	Ccl12	-3.15
NRIP2	4.35	AF357359	-3.12
TRIB3	4.28	Rian	-3.11
TRIM66	4.21	NREP	-3.08
SLC7A3	4.12	MS4A4A	-3.06
ANGPTL6	4.11	AIF1	-3.01
PPEF1	4.06	CKB	-2.99
PDZD7	3.93	NPR3	-2.92
DTHD1	3.92	AKAP5	-2.90
MEI4	3.79	CD52	-2.89
ADTRP	3.75	CD248	-2.88
PPP1R16B	3.72	RAB3IL1	-2.84
1700019D03Rik	3.72	C1QB	-2.83
P2RX3	3.69	C1QC	-2.78
ACOT2	3.67	meg3	-2.77
ELOVL3	3.66	SLCO4A1	-2.77
AVIL	3.64	TMEM119	-2.71
SLC4A10	3.61	Nxpe5	-2.71
SLC7A11	3.56	CD72	-2.70
CDH22	3.55	AQP5	-2.69
F5	3.35	SYNPO	-2.69
SLC6A9	3.30	Gm20744	-2.68
MEF2B	3.30	wisp2	-2.67
ADRA2A	3.29	ATP2B4	-2.65
CHAC1	3.28	LRRN2	-2.63
KCNMA1	3.27	CLDN15	-2.59
CATSPERD	3.26	EMILIN1	-2.59
Gbp8	3.26	cys1	-2.55
SLC7A5	3.25	CHST1	-2.52
TAF7L	3.23	FRMPD1	-2.51
PSPH	3.19	Kctd12b	-2.48
OTUD7A	3.18	TUBB4A	-2.48
PRR32	3.18	SMPD3	-2.48
dnah14	3.16	GPR153	-2.47
OSTN	3.09	Ms4a6c	-2.47
GADD45A	3.07	IGFBP4	-2.44
Akr1b7	3.06	GPR34	-2.44
GBX1	3.05	PEG10	-2.43
FGF21	3.00	EPS8L2	-2.42
LRRN3	2.98	MRC1	-2.41
1810010H24Rik	2.97	CCK	-2.40
B4GALNT2	2.97	VDR	-2.40

We used the PANTHER database to perform functional classification analysis. DEGs were initially grouped according to the PANTHER Protein Classification and results showed that the most represented protein categories among de-repressed genes were somehow involved in protein biosynthesis (Table 2). Accordingly, the PANTHER GO-Biological Process analysis showed a significant (FDR < 0.01) enrichment of processes related to both amino acids and protein metabolism (Fig. 3B, orange lines). Moreover, physical and functional interactions between de-repressed genes, determined by using the STRING platform with a highest confidence score of 0.9, revealed three main clusters which were representative of the three KEGG pathway: “Aminoacyl-tRNA biosynthesis”, “Biosynthesis of amino acids” and “Ribosome biogenesis” (Supplementary Fig. S3).

**Table 2:** PANTHER Protein Classes Most Represented in CFs pre-treated with 1 μM PTC-209 (pValue and FDR < 0.05).

PANTHER Protein Class	PANTHER ID	Type of regulation (+/−)	pValue	FDR
oxidoreductase	PC00176	+	0.000044	0.00315
methyltransferase	PC00155	+	0.0000805	0.0173
aminoacyl-tRNA synthetase	PC00047	+	0.000087	0.00932
oxidase	PC00175	+	0.000193	0.00831
cytoskeletal protein	PC00085	−	0.00000016	0.00000857
nucleic acid binding	PC00171	−	0.0000002	0.00000861
G-protein modulator	PC00022	−	0.000000241	0.00000865
defence/immunity protein	PC00090	−	0.000000454	0.0000122
enzyme modulator	PC00095	−	0.0000016	0.0000376
glycosyltransferase	PC00111	−	0.000024	0.000471
signalling molecule	PC00207	−	0.000609	0.00818
cytokine	PC00083	−	0.00075	0.00896
microtubule family cytoskeletal protein	PC00157	−	0.00137	0.0128
chemokine	PC00074	−	0.0019	0.0141
receptor	PC00197	−	0.00235	0.0168
extracellular matrix protein	PC00102	−	0.00611	0.0398

Interestingly, among de-repressed genes there were also 26 transcription factors or DNA-binding proteins (Fig. 3C), some of which playing pivotal roles in heart development. As the case of Iroquois homeobox gene 1 (Irx1), encoding a cardiac transcription factor important for the development of ventricular conduction system^20^; Krüppel-like factor 5 (Klf5), a zinc finger-containing transcription factor involved in many different cellular processes, ranging from the governance of pluripotency of embryonic stem cell to regulation of cardiovascular pathophysiology^21–23^; the transcriptional modulator Cited2, involved in Brachyury, Mesp1, Isl1, Gata4 and Tbx5 expression during cardiac differentiation of embryonic stem cells^24^; Nuclear receptor subfamily 4, group A, member 2 (NR4A2), also known as Nurr1, a member of the NR4A orphan nucleus receptor family involved in the immediate early response to different stress-stimuli, with some roles in cardiac remodelling^25^; Tbx6, a member of the evolutionarily conserved T-box family of transcription factors that are essential regulators of normal embryonic development, critical for mesoderm induction and subsequent lineage diversification by regulation of Nkx2-5 expression^26^; JMJD2A/KDM4A, a member of the JmjC domain–containing family JMJD2 of histone demethylases that catalyse the demethylation of trimethylated H3K9 (H3K9me3) and H3K36 (H3K36me3), involved in promotion of cardiac hypertrophy^27^, as well as the c-Jun dimerization protein 2, JDP2, member of the basic leucine zipper (bZIP) superfamily which typically suppresses transcription through binding to CRE and TRE DNA promoter elements and recruiting histone deacetylases^28^; Rp58 (also known Znf238, Zfp238, Zbtb18), a sequence-specific transcriptional repressor which inhibits Id genes (Id1-4) playing a central and evolutionarily conserved role during muscle formation^29^; the ubiquitous protein PHF10, a subunit of the PBAF chromatin-remodelling complexes which plays crucial role in antagonizing Polycomb action during development^30^; the forkhead box transcription factors Foxd1, which has been recently described as a promoter of iPSC generation^31^; the myocyte-specific enhancer factor MEF2b^32^.

Concerning repressed genes, they not only represent the largest group in our data set, but are also composed of heterogeneous protein subtypes. They can be grouped in a wide spectrum of protein classes involved in a broad array of processes (Table 2). Nevertheless, many targets down-regulated upon Bmi1 inhibition are involved in cell signalling. Indeed, according to the PANTHER classification, GO Biological Processes analysis revealed that many genes downregulated upon PTC-209 pre-treatment play some roles in signal transduction (Fig. 3D, yellow lines). KEGG pathway analysis in Table 3 suggests that different signal transduction pathways might be de-regulated upon Bmi1 inhibition. Many genes, indeed, seems to be involved in Wnt, Hippo, Rap1, PI3K/AKT, cAMP and calcium-dependent signalling pathways. Interestingly, 8 genes belong to the Notch pathway (Fig. 3E), which has been previously identified as down-regulated upon PTC-209 pre-treatment in leukaemia cells^33^. Moreover, we found 30 genes involved in chemokine and cytokine signalling pathways and 9 genes specific of interleukins pathway (Fig. 3E).

**Table 3:** Significantly enriched KEGG pathways (p < 0.05) – downregulated genes.

KEGG ID	Pathway Description	Genes	pValue
mmu04512	ECM-receptor interaction	14	0.0003
mmu04360	Axon guidance	17	0.0004
mmu04540	Gap junction	13	0.0008
mmu04310	Wnt signaling pathway	17	0.0012
mmu04611	Platelet activation	16	0.0015
mmu00532	Glycosaminoglycan biosynthesis - chondroitin sulfate / dermatan sulfate	6	0.0022
mmu04390	Hippo signaling pathway	17	0.0024
mmu05150	Staphylococcus aureus infection	9	0.0025
mmu04020	Calcium signaling pathway	19	0.0026
mmu04550	Signaling pathways regulating pluripotency of stem cells	16	0.0026
mmu04015	Rap1 signaling pathway	21	0.0034
mmu05217	Basal cell carcinoma	9	0.0040
mmu05412	Arrhythmogenic right ventricular cardiomyopathy (ARVC)	10	0.0046
mmu00533	Glycosaminoglycan biosynthesis - keratan sulfate	5	0.0048
mmu04924	Renin secretion	10	0.0068
mmu04713	Circadian entrainment	12	0.0073
mmu04916	Melanogenesis	12	0.0079
mmu04330	Notch signaling pathway	8	0.0086
mmu05205	Proteoglycans in cancer	19	0.0090
mmu05146	Amoebiasis	13	0.0105
mmu04151	PI3K-Akt signaling pathway	28	0.0106
mmu04510	Focal adhesion	19	0.0109
mmu04670	Leukocyte transendothelial migration	13	0.0112
mmu04514	Cell adhesion molecules (CAMs)	16	0.0113
mmu04062	Chemokine signaling pathway	18	0.0135
mmu04024	cAMP signaling pathway	18	0.0142
mmu05200	Pathways in cancer	30	0.0151
mmu01100	Metabolic pathways	78	0.0161
mmu05414	Dilated cardiomyopathy	10	0.0180
mmu04060	Cytokine-cytokine receptor interaction	20	0.0244
mmu05166	HTLV-I infection	22	0.0253
mmu04974	Protein digestion and absorption	10	0.0254
mmu05133	Pertussis	9	0.0255
mmu04610	Complement and coagulation cascades	9	0.0293
mmu04970	Salivary secretion	9	0.0314
mmu00604	Glycosphingolipid biosynthesis - ganglio series	4	0.0329
mmu05410	Hypertrophic cardiomyopathy (HCM)	9	0.0359
mmu04261	Adrenergic signaling in cardiomyocytes	13	0.0411

These subsets of genes attracted our attention, because down-regulation of inflammatory signalling has been recently related to increasing reprogramming efficiency. Muraoka *et al*., indeed, demonstrated that diclofenac sodium treatment greatly enhanced GMT-dependent cardiac reprogramming in postnatal and adult fibroblasts, but not in MEFs, via the inhibition of cyclooxygenase-2 /prostaglandin E2/PGE receptor 4/interleukin 1β/interleukin 1 receptor type 1 signalling and subsequent suppression of inflammatory and fibroblast gene programs^34^. Previous report also identified ZNF281 transcription factor as a robust and efficient activator of adult DCR through its association with Gata4 by inhibiting inflammatory signalling^35^. Moreover, there is growing evidence that innate immunity is a crucial regulator of tissue healing in neonatal hearts^36^. Indeed, a very recent paper demonstrate that CD4 + regulatory T-cells promote neonatal heart regeneration through the releasing of paracrine regenerative factors^37^.

On these bases, we performed qPCR experiment which confirmed the results of RNA-seq for the most relevant down-regulated cytokines (Supplementary Fig. S4). We also checked for the expression of other relevant cytokines and their cognate receptors which have been demonstrated to play a direct role in regulation of neonatal cardiomyocyte proliferation. Results indicate that these cytokines are not expressed in our cells (IFNγ, IFNβ, IL10) or are unmodified upon the treatment (IL1b, TNFRS1A, IFNAR1). Only in the case of TNFα a slight increase was observed that however did not reach statistical significance (Supplementary Fig. S4).

All these data prompted us to investigate whether Bmi1 inhibition could lead to a decreasing in pro-inflammatory pathway activation.

### PTC-209 pre-treatment lowers the levels of activated STAT3 and ERK1 in CFs

Down-regulation of different cytokine, chemokine and interleukin receptors, as well as of their pathway mediators, suggested us that these proteins could be at least in part responsible for the enhancement of reprogramming efficiency.

The signal transduction via type I and II cytokine receptors is based on JAKs, which in turn activates the STATs family of transcription factors, resulting in their phosphorylation and subsequent nuclear translocation^38,39^. RNA-seq clearly showed the down-regulation of different members of IL6 signalling pathway, which usually exerts its function through the activation of JAK/STAT3.

The possibility that STAT signalling could be downregulated as a secondary effect of PTC-209 activity was newsworthy, considering that the rate of DCR was increased by addition of JAK inhibitors to a combination of miRNAs 1, 133, 208, and 499^8^.

On these bases, we decided to assess the activation and subcellular localization of STAT3 in both MEFs and CFs upon 24 h PTC-209 pre-treatment.

**Figure 4:**
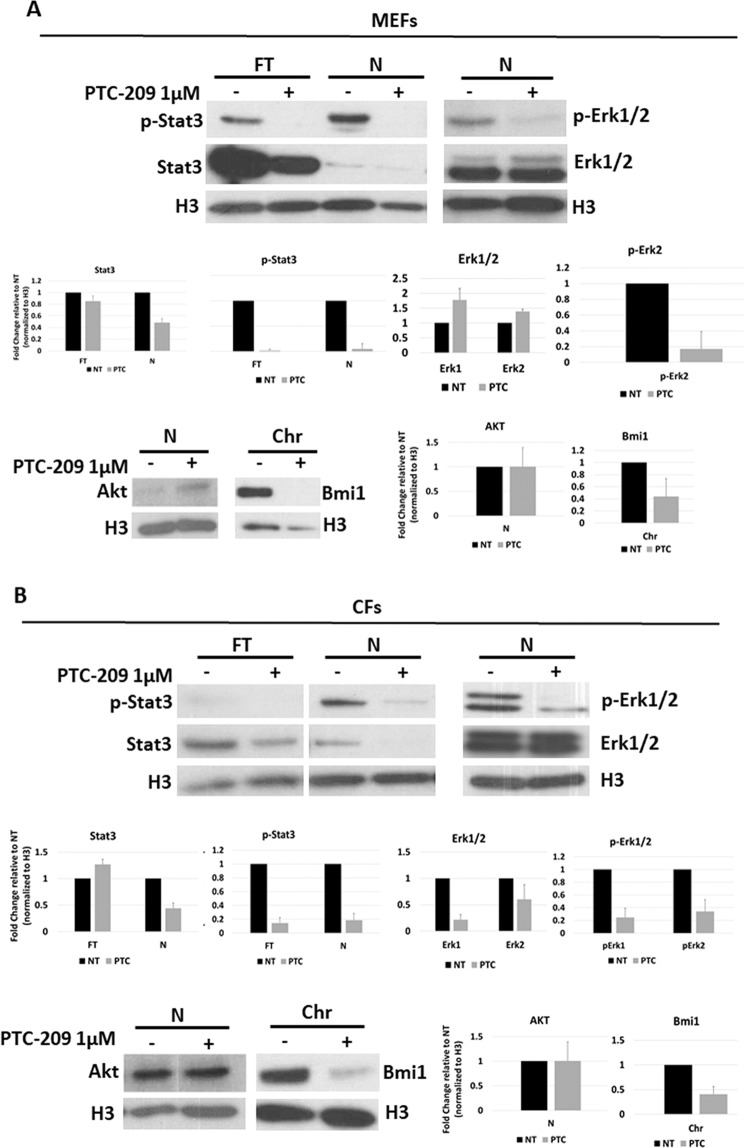
Western blot analysis showing p-STAT3, STAT3, p-ERK1/2, ERK1/2 and AKT expression in MEFs (**A**) or CFs (**B**) upon treatment with 1 μM PTC-209 for 24 h (PTC), compared to DMSO-treated (NT) cells. Histone H3 was used as loading control. Cells were fractioned into total (FT), total nuclear (N), and chromatin (Chr) fractions. Equal amounts of each fraction were loaded to allow for comparison of band intensity. Graphs report densitometric analysis of western blot on p-STAT3, STAT3, p-ERK1/2, ERK1/2, Bmi1 and AKT proteins normalized to the level of H3 loading control. Densitometric analysis of the bands was carried out using Image Pro Plus software (Media Cybernetics Inc. Rockville, MD, USA). Intensity of bands from the protein of interest were normalized to the intensity of H3 bands of the respective blots. Data are presented as means ± SD of the densitometric analysis of n = 2 blots.

As shown in Fig. 4, STAT3 total expression (FT fractions) seems to be only mildly affected by PTC-209 treatment in both MEFs (Fig. 4A) or CFs (Fig. 4B). This is in line with RNA-seq data, which didn’t show any direct modification in STAT3 gene expression. However, looking at STAT3 phosphorylation and subcellular localization it is glaring that both total (FT) and nuclear (N) fractions show a dramatic decrease of activated STAT3, suggesting a down-regulation of the pathway.

Apart from JAK/STATs, the binding of cytokines to their specific receptors may induce the activation of other signalling pathways, including the Ras-mitogen-activated protein kinase (MAPK) and phosphatidylinositol 3-kinase (PI3K) pathways^40,41^.

As shown in Fig. 4B, while AKT expression was unaffected, CFs showed a dramatic decrease of phosphorylated ERK1/2 upon Bmi1 inhibition, with ERK1 activation being more affected than ERK2. Interestingly, MEFs showed a downregulation of activated ERK2 upon 24 h PTC-209 treatment. On the contrary, AKT appeared mildly activated by PTC-209.

These data sustain the hypothesis that Bmi1 inhibition promotes CiDCR of CFs by repressing inflammatory pathways.

## Discussion

To achieve the full potential for DCR without the use of transgenes and to project cardiac reprogramming to a preclinical stage, a deeper knowledge of signalling networks that determine cell fate conversion is required to select new combinations of small molecules capable of governing CiDCR in a more efficient way^42^.

In this study we report that 24 h pharmacological inhibition of Bmi1 by 1 μM PTC-209 increases the conversion of MEFs and CFs into iCM, promoting cardiac gene expression and spontaneous beatings. Although PTC-209 was removed at the time of CRFVPT cocktail administration to the cells, it is likely that the epigenetic perturbation induced by Bmi1 inhibition is sufficient to imprint some changes in gene expression, capable to improve the effect of reprogramming cocktail. On the contrary, we found that the addition of PTC-209 for the entire reprogramming protocol resulted in a dramatic decrease in CiDCR efficacy. Probably, prolonged and powerful inhibition of Bmi1 expression could represent a barrier to the effect of CRFVPT cocktail, possibly due to some roles exerted by Bmi1 in cells undergoing trans-differentiation in later phases of reprogramming. Indeed, Bmi1 expression levels increase during CiDCR, supporting such hypothesis. Bmi1 was previously found necessary for reprogramming iPSC^43^, and Bmi1 expression is one of the shared features of many adult stem cell compartments^44^. It is thus logical to hypothesize that Bmi1 expression might be relevant for maintaining some precursor cells arising during trans-differentiation.

As PTC-209 downregulates the Bmi1 protein levels, it should faithfully recapitulate the effect of Bmi1 downregulation. Indeed, our data obtained in MEFs confirmed previous reports^18^ indicating that Bmi1 acts as an epigenetic barrier to DCR in MEFs by repressing cardiogenic loci, as Gata4 and Isl1. These events occurred together with the induction of other cardiac precursor marker genes, such as Flk1 and the down-regulation of fibroblast marker genes, such as Col1A1.

However, despite an overall improvement of CiDCR, some differences were observed between MEFs and CFs in their response to PTC-209, like no changes in cardiogenic genes in CFs upon PTC-209 administration. These prompted us to address the transcriptional effects of PTC-209 on CFs by RNA-sequence profiling and to relate them to its pro-reprogramming activity. Bioinformatic analysis of de-repressed genes showed a significant enrichment in genes involved in different steps of protein biosynthesis. This suggests that PTC-209 treatment at the dose of 1 μM, does not inhibit, rather promote active cell metabolism. After all, our data set didn’t show a significant up-regulation of the Bmi1 downstream effectors p16Ink4a, p19Arf and p53, which mediate Bmi1-dependent regulation of cell proliferation and senescence^45^. Rather, among de-repressed genes we found transcription factors playing different role in cardiovascular tissue development and cardiac differentiation.

Repressed genes, on the other hand, represent a large group of proteins mostly implicated in signal transduction pathways, whose inhibition has been suggested to be a booster for cardiac trans-differentiation, such as Wnt^10^, Hippo^46^, cAMP and calcium-dependent signalling pathways^34^ and Notch^47^. The eight genes belonging to the Notch pathway represent also an internal control, as it has been previously described that this pathway is down-regulated upon PTC-209 treatment in leukaemia cells^33^. Interestingly, Olson and colleagues showed that Notch inhibition and Akt1 activation in a cooperative manner boosted the efficiency of reprogramming of MEFs by GHMT factors up to 70%, while 45% of the generated cardiomyocytes showed spontaneous beating^47^.

It is well demonstrated that the silencing of fibroblast signatures is a prerequisite for reprogramming^17,48^. In the same line of reasoning, anti-inflammation may represent a potential target for lineage conversions associated with aging, as in case of heart regeneration upon MI^49^. Indeed, we found an overall repression of receptors and intracellular mediators of inflammation and immune response, which usually exert their function by induction of pro-inflammatory responses dependent on JAK/STAT3 and ERK1/2 activation.

This is of great interest, considering that reprogramming toward induced pluripotent stem cells (iPSCs) by overexpressing Yamanaka factors can be shifted toward cardiogenesis in the presence of a JAK inhibitor and cardiomyocyte-favourable culture condition^50^, and that the addition of JAK I inhibitor enhances miRNA-mediated reprogramming by 10-fold^8^.

Our results demonstrated that upon Bmi1 inhibition in CFs, a dramatic downregulation of both STAT3 and ERK1 nuclear translocation occurred. Of notes, the use of ERK1 inhibitors, in combination with other 8 different compounds in part overlapping CRFVPT cocktails, has been demonstrated to increase the yield of iCMs from human fibroblasts^13^.

A very recent paper described the ability of a combination of four chemicals, named IMAP, consisting of Insulin-like growth factor-1, Mll1 inhibitor MM589, transforming growth factor-β inhibitor A83-01, and PTC-209, to increase the rate of GMT-induced cardiac reprogramming by suppressing specific C-C chemokine signalling pathways^51^. These results and our findings strongly indicate that fibroblast trans-differentiation into cardiomyocytes, either obtained by transducing transcription factors or by chemical compounds, is highly enhanced by suppressing inflammatory pathways.

In conclusion, our data demonstrate for the first time that CRFVPT cocktail efficacy can be increased by 24 h pharmacological inhibition of the epigenetic modulator Bmi1, whose sole repression is sufficient to enhance CiDCR from both MEFs and CFs. We confirmed that in MEFs Bmi1 repression resulted in a major loss of H2AK119ub at specific cardiac loci, with the consequent de-repression of cardiogenic genes expression. In CFs the inhibition of Bmi1 expression correlate with repression of two major pathways related to inflammation, such as JAK/STAT3 and MAPK/ERK1/2. This is in line with previous reports indicating that inflammation and fibrosis, with their related genes, highly expressed in postnatal and adult fibroblasts compared with embryonic fibroblasts, represent age-related barriers to cardiac reprogramming^35^.

Our results may contribute to a deeper knowledge of signalling networks that determine cell fate, laying the foundations to set up new combinations of small molecules capable of governing DCR in a more efficient way.

These findings might have significant implications for the advance of new cardiac regeneration therapies.

## Methods

### Animals

All animal procedures were performed with the approval of O.P.B.A. of University of Naples Federico II. All methods were performed in accordance with the relevant guidelines and regulations.

### Cell culture

MEFs were isolated from embryonic day 13.5 (E13.5) C57BL/6 mouse embryos. The head, limbs, and internal organs were carefully removed from embryos, and then the rest tissues were washed in PBS, minced and digested with 0.05% Trypsin-EDTA into single-cell suspensions. Cells were resuspended in fibroblasts medium, consisting of DMEM (Gibco), 15% FBS (Hyclone), 2 mM Glutamax (Gibco), 0.1 mM non-essential amino acids (NEAA; Gibco), 100 units/ml penicillin and 100 μg/ml streptomycin (Gibco), and plated onto one 10 cm dish per embryo. Cells were passaged at the ratio of 1:3 (passage 1). Passage 3 MEFs were used for reprogramming

CFs were isolated from 4–6 weeks old C57BL/6 mice. Briefly, heart tissue was isolated, washed in PBS, minced and digested with a solution containing type II collagenase (3 mg/ml) and BSA (10 mg/ml) up to 45 minutes at 37 °C. CFs from three hearts were plated onto a 10 cm dish with fibroblast growth medium. Passage 1 CFs were used for reprogramming.

### Direct reprogramming of fibroblasts into iCM

The protocol of DCR was similar to previous studies with minor optimization^11^. MEFs or CFs were seeded onto six-well plates (coated with 1:100 Matrigel from BD Biosciences for 1 h at room temperature) at a density of 50 000 cells per well and cultured in fibroblast growth medium for 24 h. For PTC pre-treated samples, 1 μM PTC-209 (Sigma) was added to fibroblasts medium for 24 h. For NT samples, equal amount of DMSO was added.

After 24 h, the medium was replaced with CRM, composed of knockout DMEM (Gibco), 15% FBS, and 5% KSR (Gibco), 0.5% N2 (Gibco), 2% B27 (Gibco), 1% Glutamax, 1% NEAA, 0.1 mM β-mercaptoethanol (Gibco), 50 μg/ml 2-phospho-L-ascorbic acid (vitamin C, Sigma), 100 units/ml penicillin and 100 μg/ml streptomycin, plus CRFVPT cocktails (10 μM CHIR99021 (C, Sigma); 10 μM RepSox (R, Selleckchem); 50 μM Forskolin (F, Selleckchem); 0.5 mM VPA (V, Sigma); 5 μM Parnate, (P, Selleckchem); 1 μM TTNPB (T, Selleckchem). CRM containing chemical compounds was changed every 4 days. At the second stage of induction (day 16 for MEFs, day 20 for CFs), cells were cultured in CMM (DMEM medium with 15% FBS, 2i (3 μM CHIR99021 and 1 μM PD0325901, Sigma), 1 000 units/ml LIF, 50 μg/ml vitamin C, and 1 μg/ml insulin (Sigma)).

### Immunofluorescent staining

Cells were fixed in 4% PFA at room temperature for 30 min and washed with PBS twice. Cells were then treated with 10% FBS, 5% BSA and 0.2% Triton X-100 in PBS for 15 min, allowing blocking and permeabilization. Incubation with various primary antibodies was overnight, 4 °C. Following primary antibody incubation and washes in 1X PBS, the cells were incubated with the Alexa-Fluor 488 or 594 secondary antibodies (1:400, Molecular Probes) and nuclei were stained with Dapi (1:5000, Calbiochem). Cells were visualized with a 10×/0.30 or 20×/0.40 (dry lens) objective using inverted microscopes (DMI4000 or THUNDER Imager 3D, Leica Microsystems). The images were captured with a digital camera (DFC365 FX, Leica Microsystems) using LAS-AF software (Leica Microsystems).

Antibodies used in this study are as following: cTNT (MA5-12960, Thermo Fisher Scientific), GATA4 (SC-25310 Santa Cruz), α-MHC (MF20) (14-6503-82, eBioscience), DDR2 (sc-81707, Santa Cruz), CD31 (e-ab-30820, Elabscience), MLC2v (ab79935, Abcam), α-Actinin (A7811, Merck).

### RNA isolation and quantitative PCR

RNA isolation and quantitative PCR (qPCR) was performed as described before^52^. qPCR was carried out with the QuantStudio 7 Flex (Thermo Fisher Scientific) using Fast SYBR Green PCR Master Mix (Thermo Fisher Scientific). The housekeeping Actin mRNA was used as an internal standard for normalization. Gene-specific primers used for amplification are listed in Supplemental Table S2^53^. qPCR data are presented as fold changes relative to the indicated reference sample using 2DeltaCt comparative analysis^54^.

### Preparation of cell extracts, SDS-PAGE and Western blot analysis

Fractionated extracts were obtained as described before^55^. Briefly, cells were trypsinised, counted, washed with 5 ml of PBS, pelleted, resuspended in 10x of packed cell volume (usually 500 µl) of ice-cold buffer A (10 mM HEPES [pH 7.9], 10 mM KCl, 1.5 mM MgCl2, 0.34 M sucrose, 10% glycerol, 1 mM DTT, 0.1% Triton X-100, protease inhibitors), and incubated 10 min on ice. An aliquot (50 µl) of the total fraction was taken (Fraction T). After incubation, samples were centrifuged at 1,300 x g for 5 min at 4 °C. The pellet was washed with 5x volumes buffer A and then resuspended in 1x volume buffer A. An aliquot (5 µl) for the total nuclear fraction was taken (fraction N). The sample was then diluted in 10x volume of buffer B (3 mM EDTA, 0.2 mM EGTA, 1 mM DTT, and protease inhibitors), briefly vortexed, and incubated for 30 min on ice. Samples were centrifuged at 1,700 x g for 5 min at 4 °C. Pellets were washed in 5x volume buffer B and then resuspended in 10x volume B-SDS 1x lysis buffer (50 mM Tris-Cl [pH 7.5], 2 mM EDTA, 2% SDS) to form the chromatin fraction (fraction Chr). All fractions (except the chromatin fraction) were then mixed with an equal amount of B-SDS 2x lysis buffer, and all were boiled for 10 min. The chromatin fraction was also sonicated for 5 min at high potency (30 s ON, 30 s OFF) until clarified.

For total extracts, cells were lysed with lysis buffer containing 10 mM Tris-HCl pH 7,4, 150 mM NaCl, 1 mM EDTA pH 8, 1% Triton X-100 supplemented with cocktail protease inhibitors (Roche Diagnostics, Indianapolis, USA). Protein concentration was determined by Bradford assay (Bio-Rad, USA).

Western blot was performed as described before^56^, using primary antibody for Bmi1 (rabbit monoclonal, D42B3, Cell Signaling), p-Stat3 (Y705) (rabbit monoclonal, D3A7, Cell Signaling), Stat3 (mouse monoclonal, 124H6, Cell Signaling), Akt (rabbit, 9272 S, Cell Signaling), H3 (rabbit, 06-755, Millipore), pErk1/2 (p-p44/42 Mapk T202/Y204) (rabbit monoclonal, D13.14.4E, Cell Signaling), Erk1/2 (rabbit polyclonal, sc-94, Santa Cruz), Actin (Sigma) overnight at 4 °C. Blots were washed with TBST for three times and further incubated for 1 h with secondary antibodies HRP-conjugated goat anti-rabbit (Santa Cruz, USA sc-2004) and anti-mouse IgG (Santa Cruz, USA, sc-2005). Bound antibodies were detected by the ECL system (Santa Cruz, USA, sc-2048). Densitometric analysis was performed using ImageJ 1.52 v software.

### Chromatin immunoprecipitation-qPCR analysis

Chromatin immunoprecipitation (ChIP) assays were performed as described before^57^, optimized for fibroblast cells.

Briefly, cells were cross-linked with 1% formaldehyde for 10 min at room temperature; the reaction was then quenched by adding glycine at a final concentration of 125 mM. The chromatin was sonicated to an average DNA fragment length of 500 to 1000 bp. Soluble chromatin extracts were immunoprecipitated using 3 to 5 µg of Ubiquityl-Histone H2A (Lys119) (D27C4) XP® (BK8240S, Cell Signaling). Appropriate IgGs (Abcam) were used as a negative control. Supernatant obtained without antibody was used as an input control. After qPCR, the results were analysed using an average of Ct of no antibody and IgG as background. The 2ΔCt of each sample was related to the 2ΔCt of the input sample. The percentage of total chromatin was calculated as 2ΔCt × 10, where ΔCt = Ct (input)–Ct (IP). Oligonucleotide pairs used have been previously described^18^ and are listed in Supplemental Table S2.

### Fluorescence-activated cell sorting analysis

Quantitative analysis of iCM was performed by flow cytometry. Briefly, reprogrammed cells were dissociated with incubation in 0.05% Trypsin/EDTA at 37 °C for 10 min, washed in PBS, and resuspended in a blocking and permeabilizing solution containing 2% FBS, 5% BSA and 0.2% Triton X-100 in PBS for 30 min. Incubation with primary antibody α-MHC (MF20) (14-6503-82, eBioscience) was performed overnight at 4 °C. Following three washes in 1X PBS, cells were incubated with the Alexa-Fluor 488 secondary antibodies (1:400, Molecular Probes) for 30 minutes in the dark, and then analysed with a Fluorescence-Activated Cell Sorting (FACS) flow cytometer Accuri C6 (Becton Dickinson; San Diego, CA, USA).

### RNA-sequencing

The RNA-seq library preparation and libraries sequencing were performed by the Next Generation Sequencing Core Facility (LaBSSAH) of CIBIO – University of Trento. The sequencing data were uploaded to the Galaxy web platform, and we used the public server at usegalaxy.org to analyse the data^58^. Sequences alignment to reference genome was performed by using the RNA STAR tool. Differential gene expression analysis was done using the R package DESeq 2. The abundance of genes was used to calculate fold change and p values. Cut off values of fold change greater than 2 and p Adjusted values less than 0.05 were then used to select for differentially expressed genes between sample group comparisons.

Significant pathway enrichment analysis, Biological processes and Protein classes was performed using PANTHER Overrepresentation Test^59^.

Search Tool for the Retrieval of Interacting Genes/Proteins (STRING) was used to investigate the predicted gene–gene interaction network^60^.

### Statistical analysis

The number of biological replicates of each experiment is indicated in the figure legends. The means of at least 3 independent experiments were used to calculate SEM or SD and to perform statistical analysis (when appropriate). All P values were calculated by Student’s t test.

## Supplementary information

Supplementary Figures.

Supplementary Tables.

Supplementary movie S1.

Supplementary movie S2.

## Data Availability

The datasets generated during and/or analysed during the current study are available from the corresponding author on reasonable request.
