# Supplementary material for: Bmi1 inhibitor PTC-209 promotes Chemically-induced Direct Cardiac Reprogramming of cardiac fibroblasts into cardiomyocytes
Source: Sci Rep. 2020 Apr 28;10:7129. doi: 10.1038/s41598-020-63992-8 (PMC7189257; doi:10.1038/s41598-020-63992-8)
Supplement: Supplementary file 1 — Supplementary Figures. [file 41598_2020_63992_MOESM1_ESM.pdf]

Supplementary Information

Bmi1 inhibitor PTC-209 promotes Chemically-induced Direct Cardiac Reprogramming of cardiac fibroblasts into cardiomyocytes

Gianluca Testa[1], Michele Russo[2], Giorgia Di Benedetto[3], Matteo Barbato[3], Silvia Parisi[3], Flora Pirozzi[2], Carlo Gabriele Tocchetti[2], Pasquale Abete[2], Domenico Bonaduce[2], Tommaso Russo[3], Fabiana Passaro[3*].

Author Affiliations

[1]Department of Medicine and Health Sciences "Vincenzo Tiberio", University of Molise, Via Francesco de Sanctis 1, 86100 - Campobasso, Italy; [2]Department of Translational Medical Sciences; University of Naples "Federico II", Via Sergio Pansini 5, 80131 – Napoli, Italy; [3]Department of Molecular Medicine and Medical Biotechnology; University of Naples "Federico II", Via Sergio Pansini 5, 80131 – Napoli, Italy.
Correspondence and requests for materials should be addressed to Fabiana Passaro (email: fabiana.passaro@unina.it).

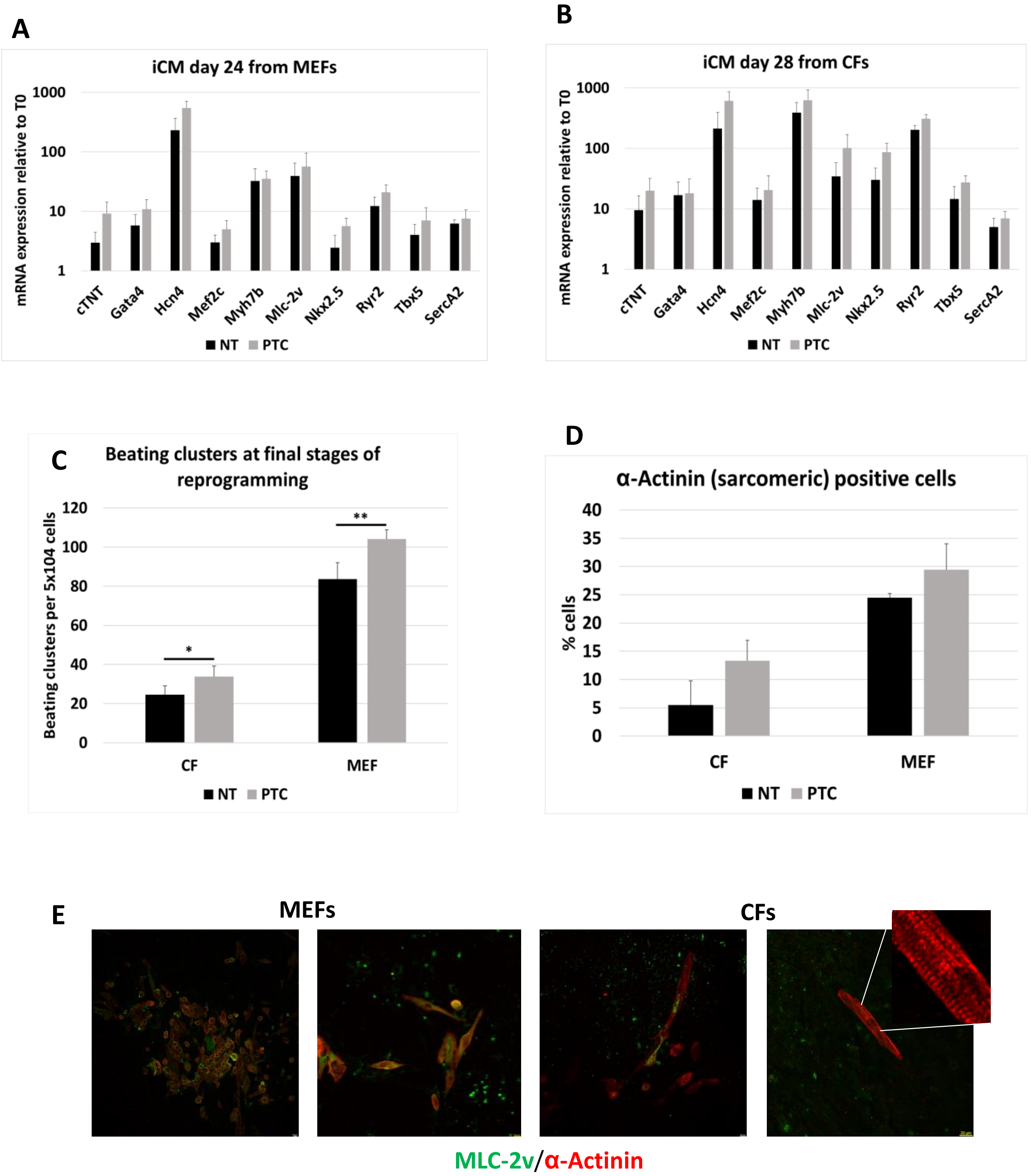

Figure S1. A-B) qRT-PCR analysis of cardiac lineage markers: cTNT, Gata4, Hcn4, Mef2c, Myh-7b, Mlc-2v, Nkx2.5, Ryr2, Tbx5 and SercA2 at final stage of CiDCR, starting from MEFs (A) and CFs (B). Data represent mRNA expression levels relative to T0. RNA was collected from the whole cell population present in the plate at day 24 (MEFs) or 28 (CFs). Data are Means ± SEM of a representative experiment, n=2. **C)** Number of beating clusters of iCM from MEFs or CF pre-treated with 1 µM PTC-209 (PTC) or DMSO (NT) at final stages of CiDCR. Results are presented as means ± SEM, n = 4. * = $p<0.05$; ** = $p<0.01$. **D)** Percentage of α-actinin positive cells with assembled sarcomeres at final stages of CiDCR upon PTC-209 pre-tratment. The histograms represent the percentage of α-actinin-positive cells counted in more than 4 independent fields, per total cells. **E)** Representative staining of α-actinin-positive cells. Scale bars: 20 µM.

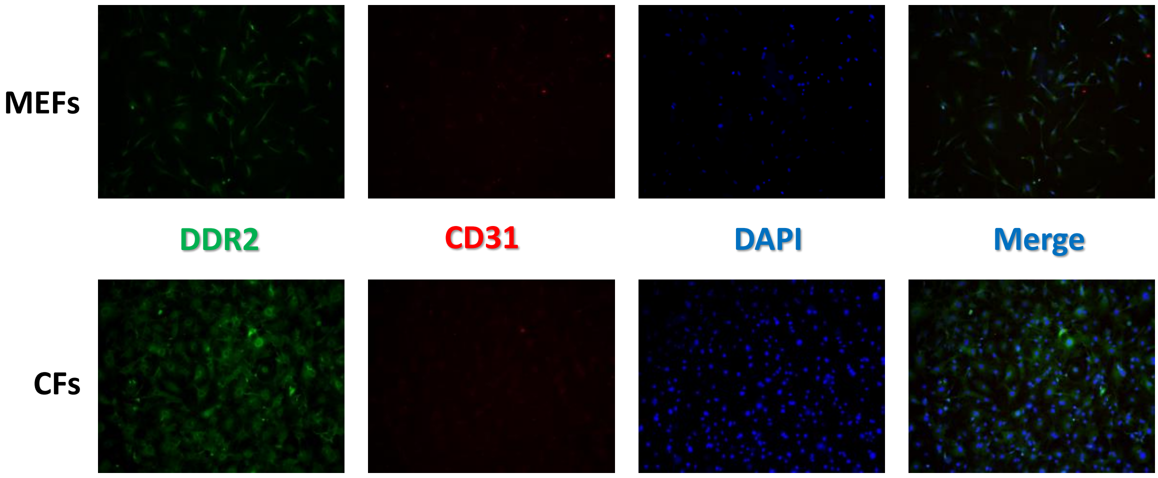

Figure S2. Representative immunostaining of MEFs and CFs for the fibroblasts marker Discoidin Domain Receptor 2 (DDR2) and the endothelial marker platelet-endothelial cell adhesion molecule 1 (PECAM1/CD31). Images are related to MEFs at passage 2, isolated from 13.5 dpc C57BL embryos, and to CFs at passage 2, isolated from 5 weeks old C57BL mice. Scale bars: 40 µM.

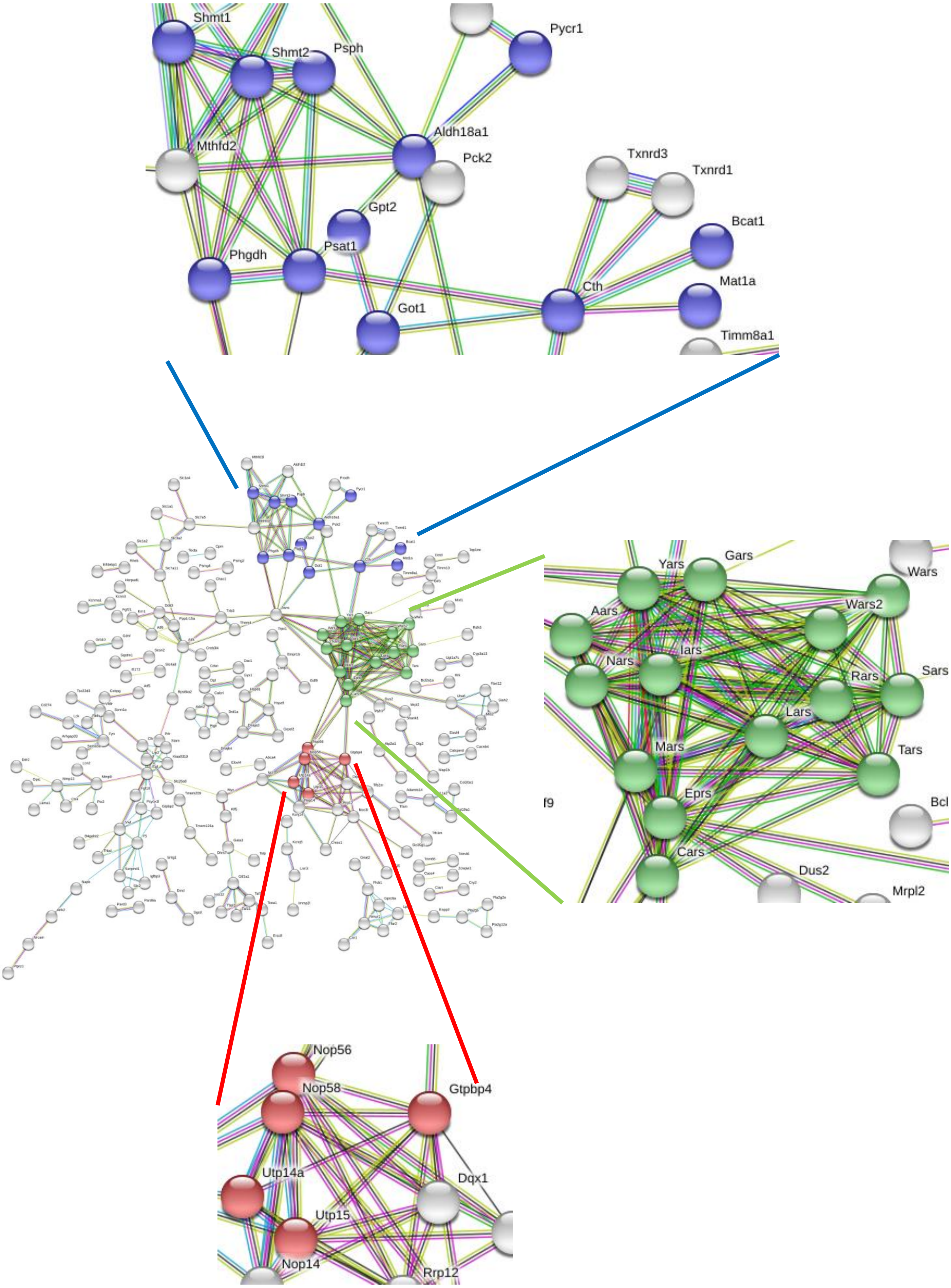

KEGG ID	PATHWAY DESCRIPTION	FDR
mmu00970	Aminoacyl-tRNA biosynthesis	0.0143
mmu01230	Biosynthesis of amino acids	0.0055
mmu03008	Ribosome biogenesis	0.0055

Figure S3. Physical and functional interactions between up-regulated genes determined by using the STRING platform, with the highest confidence score of 0.9, and related KEGG pathway.

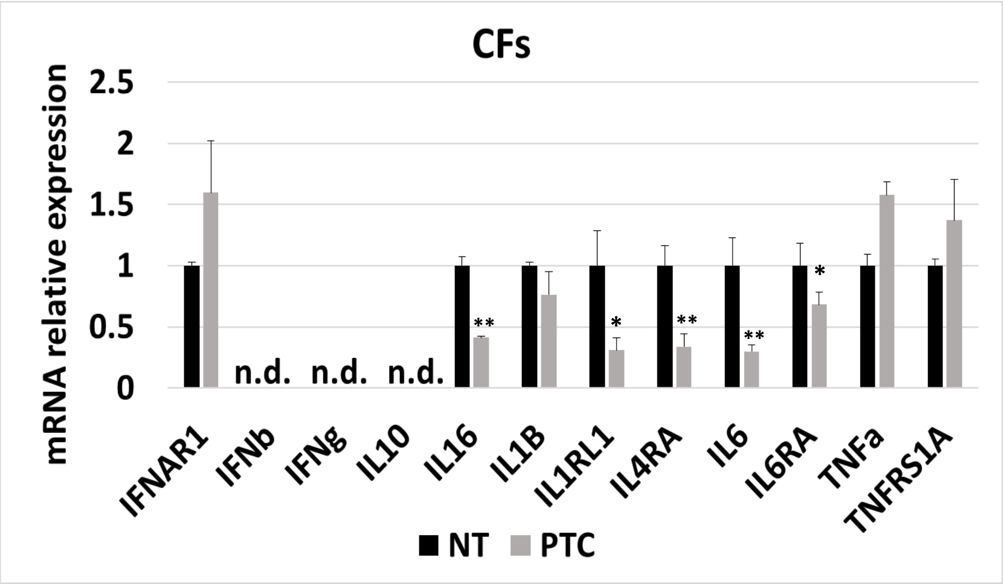

Figure S4. qRT-PCR analysis for the validation of down-regulated genes belonging to the Interleukin signalling pathway upon 24h PTC-209 pre-treatment (PTC). Data were normalized to Actin and related to DMSO treated (NT) control cells. N.D: Not detectable (mean ± SD; n = 3; *$p < 0.05$; **$p < 0.01$).
